# Expression of Kv3.1b potassium channel is widespread in macaque motor cortex pyramidal cells: A histological comparison between rat and macaque

**DOI:** 10.1002/cne.24192

**Published:** 2017-03-14

**Authors:** David Soares, Isabelle Goldrick, Roger N. Lemon, Alexander Kraskov, Linda Greensmith, Bernadett Kalmar

**Affiliations:** ^1^ Sobell Department of Motor Neuroscience and Movement DisordersUCL Institute of NeurologyQueen SquareLondonWC1N 3BGUnited Kingdom

**Keywords:** interneuron, macaque, motor cortex, Kv3.1b potassium channel, RRID:AB_2131480, RRID:AB_91735, RRID:AB_2315331, RRID:AB_10000344

## Abstract

There are substantial differences across species in the organization and function of the motor pathways. These differences extend to basic electrophysiological properties. Thus, in rat motor cortex, pyramidal cells have long duration action potentials, while in the macaque, some pyramidal neurons exhibit short duration “thin” spikes. These differences may be related to the expression of the fast potassium channel Kv3.1b, which in rat interneurons is associated with generation of thin spikes. Rat pyramidal cells typically lack these channels, while there are reports that they are present in macaque pyramids. Here we made a systematic, quantitative comparison of the Kv3.1b expression in sections from macaque and rat motor cortex, using two different antibodies (NeuroMab, Millipore). As our standard reference, we examined, in the same sections, Kv3.1b staining in parvalbumin‐positive interneurons, which show strong Kv3.1b immunoreactivity. In macaque motor cortex, a large sample of pyramidal neurons were nearly all found to express Kv3.1b in their soma membranes. These labeled neurons were identified as pyramidal based either by expression of SMI32 (a pyramidal marker), or by their shape and size, and lack of expression of parvalbumin (a marker for some classes of interneuron). Large (Betz cells), medium, and small pyramidal neurons all expressed Kv3.1b. In rat motor cortex, SMI32‐postive pyramidal neurons expressing Kv3.1b were very rare and weakly stained. Thus, there is a marked species difference in the immunoreactivity of Kv3.1b in pyramidal neurons, and this may be one of the factors explaining the pronounced electrophysiological differences between rat and macaque pyramidal neurons.

## 1 Introduction

The descending motor pathways in mammals exhibit many species‐specific differences in both their structure and their function. Descending pathways originating from the cortex arise from layer V pyramidal neurons, and include corticostriatal, corticobulbar, corticopontine, and corticospinal projections, among others. For instance, species differences in the corticospinal system include variation in the cortical areas giving rise to the tract, in the size and distribution of corticospinal neurons and their axons, in the route these axons take within the spinal cord, and in their targets within the spinal gray matter (Kuypers, 1981; Lemon, 2008; Lemon & Griffiths, 2005). Differences in the organization of motor pathways are likely to reflect the variety of different functions that they mediate in different species.

In the rat, pyramidal neurons typically have action potentials with a broad duration (typically ∼900 µs), in contrast to many fast‐spiking cortical interneurons which exhibit much shorter duration spikes (∼400 µs; Bartho et al., 2004). Differences in spike duration between interneurons and pyramidal cells in rats are partly due to different levels of expression of Na^+^ and K^+^ channels (Bean, 2007; Erisir, Lau, Rudy, & Leonard, 1999; Martina & Jonas, 1997; Martina, Schultz, Ehmke, Monyer, & Jonas, 1998; Suter, Migliore, & Shepherd, 2013). Fast‐spiking properties reflect the presence of Kv3 and Kv1 channels which allow rapid repolarization. Kv3.1b mRNA and protein are associated with fast‐spiking interneurons in rats, which express parvalbumin (Bean, 2007; Rudy & McBain, 2001). The expression of Kv3.1b in rat pyramidal neurons is generally very low (Chow et al., 1999).

In contrast to the rat, in both the cat and macaque, pyramidal neurons can exhibit action potentials of short duration (Chen, Zhang, Hu, & Wu, 1996; Lemon, Vigneswaran, Waldert, Philipp, & Kraskov, 2012; Matsumura, 1979; Takahashi, 1965). In the awake macaque, extracellular recordings in primary motor cortex from identified corticospinal neurons (which are just one subclass of pyramidal neuron), have spikes as brief as 160 µs, with a mean duration of only 260 µs (Vigneswaran, Kraskov, & Lemon, 2011). The rapid repolarization of pyramidal neurons in the macaque could, in theory, allow very high spike discharge rates.

In keeping with this finding of brief spikes in macaque pyramidal neurons, there have been several reports of Kv3.1b being expressed in layer V pyramids in macaque cortex, including large pyramids in motor cortex (Constantinople, Disney, Maffie, Rudy, & Hawken, 2009; Ichinohe et al., 2004). However, there has never been a systematic comparison of Kv3.1b expression in rat and macaque motor cortex to reveal the extent to which pyramidal cells in monkey motor cortex express Kv3.1b potassium channels, and whether the expression of these channels is markedly different from that described in the rat.

In this study, we have used two different antibodies for Kv3.1b to make a direct comparison of its expression in rat and macaque cortical neurons, using parvalbumin‐expressing interneurons as a control cell population in both species. Pyramidal neurons were identified both by their characteristic shape and by labeling with the pyramidal cell neurofilament marker SMI32. This antibody has been described to label a large proportion of layer 3 and layer 5 pyramidal cells in the cortex of several species, including rat, monkey, and human (Campbell & Morrison, 1989; Gabernet, Meskenaite, & Hepp‐Reymond, 1999; Sternberger & Sternberger, 1983; Wakabayashi, Hansen, & Masliah, 1995). We measured the intensity of Kv3.1b expression in the soma membrane of these pyramidal neurons. We confirmed that in the rat motor cortex, very few SMI32‐postive pyramidal neurons express Kv3.1b, while its expression is common among macaque motor cortex pyramidal neurons. Labeled pyramids included large (Betz) cells, but also many smaller pyramidal neurons. Our results suggest that there are major differences in the prevalence of the fast rectifying potassium channel Kv3.1b in pyramidal cells of the motor cortex in rat and macaque, which may be linked to the species‐related differences in spiking patterns and output functions of these important neurons.

## 2 Materials and methods

### 2.1 Animals and perfusion

All experiments were approved by the respective local Animal Welfare and Ethical Review Body and were carried out in accordance with the UK Animals (Scientific Procedures) Act.

#### 2.1.1 Rat

Five adult, female Wistar rats (200–300 g) were deeply anesthetized using an overdose of pentobarbital and perfused transcardially first with saline followed by 4% paraformaldehyde (PFA). The brain was dissected and postfixed in 4% PFA solution overnight at 4 °C and then cryopreserved in sucrose solution (30% in phosphate‐buffered saline; PBS) at 4 °C. A small block of forelimb motor cortex was identified from its stereotaxic coordinates (−1 caudal to + 3 mm rostral of bregma and 1 to 4 mm lateral to the midline) and removed before being cut on a cryostat at 20 μm; every third section was collected serially. A total of 60 sections were collected from each rat and stored at −20 °C for further processing.

#### 2.1.2 Monkey

Four adult macaque monkeys (*Macaca mulatta*) were used for this study. All monkeys were purpose‐bred for research and they were socially housed. One female (monkey R, 5 years old, 5.5 kg) and one male (S, 4.5 years old, 7.0 kg) had not been used in any other procedure. Two other males (monkey A, 8 years old, 7.1 kg and B, 8 years old, 8.7 kg) had earlier been used for recordings from a region of cortex remote from that sampled in this study, and contributed to other, related studies which have already been published (Lemon et al., 2012; Vigneswaran et al., 2011). Following initial sedation with an intramuscular injection of ketamine, the monkey was killed with an overdose of pentobarbital. The chest was opened and a large bolus of heparin (0.5 ml) was injected into the left ventricle. The left ventricle was then opened and a large cannula passed into the aorta and clamped there. The right auricle was opened. A perfusion pump was used to deliver a PBS prewash (1.5 L) at room temperature at a fast rate. Thereafter, the pump was switched to infuse cold fixative (3% paraformaldehyde in PBS, 3.5 L). After an initial fast perfusion, the speed was then reduced so that the full volume of fixative (3.5 L) was perfused within ∼20–30 min. In two monkeys, the entire brain was removed and placed in 3% PFA for post‐fixation. Small blocks of tissue (∼ 5 × 5 × 5 mm) were subsequently removed from the arm/hand and leg areas of the primary motor cortex (M1). The arm/hand block was from tissue 15–20 mm lateral from the midline, while the leg area block was from 0 to 5 mm lateral. The blocks were transferred into PBS containing an increasing concentration of sucrose preservative starting with 10%, then 20%, and finally 30% sucrose (24 hrs in each). In the other two monkeys, perfusion with fixative was followed by successive perfusions of 10, 20, and 40% sucrose in PBS, and the brain was then removed. Once again, blocks were cut from the arm/hand and leg areas of M1.

Serial sections from the cortical blocks were cut in the parasagittal plane on a cryostat at 20 μm; every fourth section was collected into the same analysis cohort. A total of at least 18 sections were collected for each type of analysis from each monkey and stored at −20 °C.

### 2.2 Immunohistochemistry

In order to validate our immunohistochemical analysis and the specificity of the antibodies, we investigated Kv3.1b expression using two different antibodies. First we used a mouse monoclonal antibody (NeuroMab, 75‐041, from UC Davis/NIH NeuroMab Facility, US; used at 1:100 dilution; RRID:AB_2131480) that was raised against the C terminal amino acid sequence between 437 and 585 of the human Kv3.1b protein, which is identical to the same sequence in the macaque monkey (Accession No P25122). Homology between rat (*Rattus norvegicus; NP 036988.1*) and macaque monkey (*M. mulatta NP001180751.1*) full length Kv3.1b amino acid sequence is 99.8%, with one single amino acid difference in position 237, which is outside of the area recognized by our antibodies (as analyzed using EMBL‐EBI Multiple Sequence Comparison by Log‐Expectation tool).

We also used a rabbit polyclonal antibody from Millipore (Formerly known as Chemicon, from Merck Millipore, Watford, UK; Cat No AB5188, used at 1:100; RRID:AB_91735) that was raised against the amino acid sequence between 567 and 585 of the rat Kv3.1b protein, a region exclusively present on Kv3.1b isoform and not the Kv3.1a isoform of the protein (Perney & Kaczmarek, 1997). In order to ensure we only analyzed specific Kv3.1b immunoreactivity, we tested the specificity of our antibodies using a preabsorption protocol. Both antibodies preabsorbed with the appropriate control peptides served as additional negative controls. Antibody preabsorption was carried out by incubating the primary antibody with 10x molar quantity of the relevant control peptide overnight at 4 °C. These sections processed using preabsorbed antibodies did not give any labeling above the level of autofluorescence.

For the identification of cortical pyramidal cells, we used a mouse monoclonal antibody against the nonphosphorylated neurofilament heavy chain SMI32 (Campbell & Morrison, 1989; Covance, New Jersey, US; used at 1:500; RRID:AB_2315331), and for the identification of cortical interneurons we used rabbit and mouse antibodies against the calcium‐binding protein parvalbumin (PV25; rabbit polyclonal antibody, used at 1:1,000; RRID:AB_10000344; PV235; mouse monoclonal antibody, used at 1:5,000; RRID:AB_10000343; both by Swant, Marly Centre, Switzerland).

### 2.3 Double immunofluorescence

For this study, the following double‐labeling experiments were carried out:

1. Parvalbumin (rabbit) and NeuroMab (mouse) Kv3.1b
2. Parvalbumin (mouse) and Millipore (rabbit) Kv3.1b
3. SMI32 (mouse) and Millipore (rabbit) Kv3.1b

For analysis of Kv3.1b labeling, we employed the Tyramide Signal Amplification TSA^TM^PLUS (PerkinElmer Life and Analytical Sciences, US, NEL741) system, which specifically enhances the labeling signal and enables more robust visualization of the antibody labeling. Due to the unavailability of antibodies against specific nonphosphorylated neurofilament heavy chain raised in species other than mouse, we could not carry out double labeling using the NeuroMab (mouse) Kv3.1b antibody and SMI32. In these sections, pyramidal cells were identified by their shape, size and lack of parvalbumin expression.

Sections for double immunofluorescence were first permeabilized using Tris‐buffered saline (TBS) containing 0.1% Triton‐X‐100 (TBS‐T). Sections were then incubated in 0.3% H_2_O_2_ for 2 min at room temperature in order to quench endogenous peroxidise activity. Unspecific labeling was blocked in TNB buffer (supplied with Tyramide amplification kit) supplemented with 10% normal goat serum at room temperature for 1 hr. Sections were then incubated overnight at 4 °C in primary antibodies diluted in the blocking solution. The following control sections were also prepared: (i) negative controls in which the primary antibody was omitted, and (ii) preabsorbed negative control sections, in which the antibody was preabsorbed using its control peptide. Sections were then washed three times for 5 min with TBS prior to incubation for 2 hr with the secondary antibodies diluted in TNB buffer. The secondary antibody used for the mouse Kv3.1b antibody was biotinylated goat anti‐mouse IgG (Vector, Peterborough, UK; 1:100), whereas the rabbit Kv3.1b labeled sections were incubated in a biotinylated goat anti‐rabbit IgG (Vector, Peterborough, UK; 1:100). Cell markers were visualized by incubation with the appropriate Alexa 568 labeled secondary antibody: for SMI32 and mouse parvalbumin a goat anti‐mouse IgG by Invitrogen was used at 1:1,000, whereas for the parvalbumin, a goat anti‐rabbit IgG from Invitrogen, Carlsbad, California, US, was used at 1:1,000. Following washes in TBS, sections were incubated for 1 hr in horseradish peroxidase conjugated Avidin (ABC; Vector laboratories, Peterborough, UK, PK6100). Following washes in TBS, sections were then incubated in the FITC‐labeled Fluorophore Tyramide Amplification Reagent diluted 1:200 for 45 s. After washes in TNB, sections were stained with DAPI (4′,6‐diamidino‐2‐phenylindole, nuclear marker; 1 µg/ml) and the sections were also incubated in Sudan black for 20 min to reduce autofluorescence. Sections were then mounted using fluorescent mounting medium (DakoCytomatio, Cytomation; Glostrup, Denmark; Fluorescent Mounting Medium, S3023).

### 2.4 Confocal microscope imaging

Sections were visualized using a Zeiss LSM 710 Laser Scanning Confocal Microscope attached to a Zeiss Axio Observer Microscope with a 40x and 63x oil immersion objective lens. Using the ZEN software and camera, 1024 × 1024 pixel resolution single plane images were taken from the rat motor cortex and from the rostral bank of the central sulcus in the macaque motor cortex for further analysis.

### 2.5 Quantitative analysis of Kv3.1b labeling on pyramidal cells and interneurons

**Figure 1:**
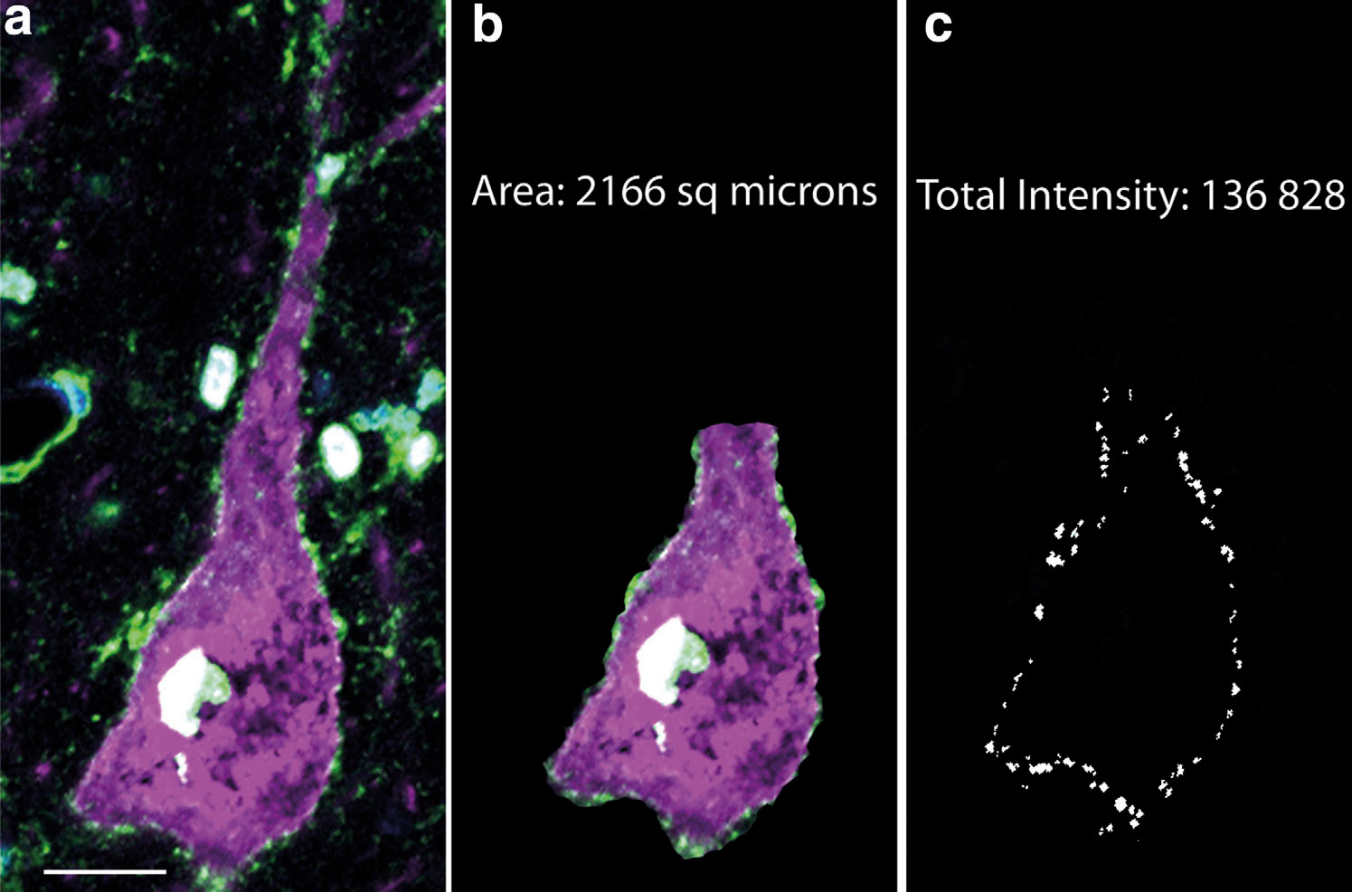
Analysis of Kv3.1b labeling in neuronal cell membranes. Sections stained for Kv3.1b were visualized and images taken using the 63x objective of a Zeiss LSM710 confocal microscope. (a) Pyramidal neuron in macaque motor cortex stained for SMI32 (magenta) and Kv3.1b Millipore antibody (green) and DAPI (blue). (b) The same neuron shown in a, indicating the area of cell soma and base of apical dendrite that were manually delineated using the Metamorph image analysis software. The cross‐sectional area was measured. This selection was color separated and thresholded. The staining of the nucleus was not included in the analysis. (c) Image mask showing the selected area of membrane. The white regions making up the mask represent all the regions of membrane labeling that were above a standard threshold value used for all images. The intensity of Kv3.1b staining underneath the mask was measured on the original image (a) and summed to provide the total intensity measure for the cell membrane of that neuron. Scale bar: 20 µm [Color figure can be viewed at wileyonlinelibrary.com]

Single plane confocal images were further analyzed using the MetaMorph Offline software (version 7.5 of Meta Imaging Series). First, cells expressing magenta cell markers (reflecting either parvalbumin (experiments 1 and 2) or SMI32 staining (experiment 3) were delineated using the “Trace Region Tool” (see Figure 1a). In cells stained for the NeuroMab Kv3.1b antibody, where SMI32 colabeling was not possible, we selected and delineated neurons displaying pyramidal cell morphology. Using the Trace Region tool, we outlined the cell membrane making up the perimeter of the cell soma and the base of the apical dendrite (Figure 1b). The area within this perimeter was taken as the cross‐sectional area of the cell body. Each image was split according to wavelength (magenta for cell markers, green for Kv3.1b and, in some cases when DAPI was used, blue). The selected cell membranes were internally thresholded to exclude very low intensity labeling that was considered unspecific. The same thresholds were applied to all images analyzed. Then, for each image viewed with green wavelength for Kv3.1b labeling, an image mask was generated that depicted the membrane areas of interest with pixels that had intensities above the threshold at that wavelength (Figure 1c). All the measured images were from cell bodies in which the nucleus was fully visible, but may not have been taken at the optimal section for maximum cross‐sectional area of the soma. Kv3.1b labeling was also present within cell nuclei, for both NMAB and Millipore antibodies. However, antibody reabsorption using a peptide against which the antibody was raised eliminated this labeling, indicating that this labeling is due to our primary antibodies. The presence of Kv3.1b at mRNA and protein levels in pyramidal cell nuclei had been described before (Perney & Kaczmarek, 1997). Since our aim was to measure Kv3.1b labeling present on neuronal membranes, we excluded cell nuclei from the measurements and only measured staining intensity on the cell membrane.

Next, Integrated Morphometry Analysis was performed, using the image mask to select, on the original image, all regions of the cell membrane that were above threshold. The total intensity of all these labeled regions was calculated on the original image. Data for each cell analyzed were collected and entered into an Excel spreadsheet. For further analysis, SMI32‐positive pyramidal cells, parvalbumin‐positive putative interneurons, and (in NeuroMab stained sections) neurons identified in terms of shape and size as putative pyramidal neurons, were treated as separate groups. Kv3.1b staining intensities were recorded separately for each group.

### 2.6 Analysis of data

For each cell analyzed, the total cross‐sectional cell area and the total intensity of Kv3.1b labeling within the selected membrane area were determined (Figure 1). Next, the intensity data set generated for each experimental condition was binned and frequency distribution graphs generated, in order to reveal staining distribution differences within the same species and compare them between different cell types (pyramidal and parvalbumin‐positive interneurons). Distribution plots were generated for log of total intensity as well as log of total intensity normalized to the perimeter of the labeled cell membrane. Here we show results using the total intensity data as the results were essentially the same for both measures. In the case of using the Millipore Kv3.1b antibody, which was co‐labeled with interneuron (parvalbumin) and pyramidal cell markers (SMI32), we also plotted Kv3.1b intensity against the measured cross‐sectional area of the soma.

## 3 Results

In order to validate the presence of Kv3.1b labeling on specific cell populations, we carried out double labeling experiments using antibodies raised against Kv3.1b and markers of pyramidal cells as well as interneurons. One class of cortical interneurons is known to be positive for the calcium‐binding protein parvalbumin, whereas many pyramidal cells are known to be positive for a hypophosphorylated form of neurofilament (SMI32).

We carried out the staining using two separate antibodies for Kv3.1b (NeuroMab and Millipore) that have been raised against different fragments of the Kv3.1b protein, that are nevertheless 100% homologous between the rat and the macaque monkey. We did this in order to show that both antibodies recognize the same, membrane bound structures on cortical neurons. The Kv3.1b protein is one of the two products of the KCNC1 gene, located on chromosome 11 in humans. This gene has two protein coding transcripts; one longer (transcript ID: ENST00000265969.6), coding for the 585 amino acid KV3.1b protein and a shorter (transcript ID: ENST00000379472.3), coding for a truncated, 511 amino acid Kv3.1a protein, which lacks 74 amino acids at its C terminal. One of our antibodies (Millipore) is specifically raised against the C terminal segment of Kv3.1b protein that is not present in Kv3.1a and thus cannot cross‐react with this isoform. Although the Neuromab antibody was raised against a longer sequence that contains some homologous sequences with Kv3.1a, the majority of the amino acid sequence used for the generation of this antibody is also exclusively present in the C terminal region of the Kv3.1b protein.

Double labeling experiments were carried out for the rabbit Kv3.1b antibody together with mouse parvalbumin and SMI32, respectively. We co–labeled this mouse Kv3.1b antibody with parvalbumin only due to the unavailability of antibodies against hypophosphorylated neurofilaments raised in species other than mouse. Therefore, we were unable to co‐label Kv3.1b using the NeuroMab antibody with the neurofilament heavy chain pyramidal cell marker. In addition to cell markers, we also used morphological criteria to identify different cell populations. Thus, interneurons were generally identified by their round shape. Many were small in size (estimated cross‐sectional area 100–400 µm^2^) and had a nucleus relatively large in comparison with the rest of soma. Pyramidal cells were identified by the presence of SMI32 labeling and also by their triangular‐shaped cell body and thick apical dendrite. The nucleus was relatively small in comparison with the rest of the soma. In order to make a comparison between rat and macaque motor cortices for the expression pattern of Kv3.1b revealed by the two different Kv3.1b antibodies used in this study, we analyzed images taken from each staining using the same analytical criteria, such as thresholding levels and measurements of staining intensities as described in Methods. For both antibodies, we observed specific labeling present in cell membranes as well as in nuclei of some but not all cells in the motor cortex sections of rat and macaque monkey. Although the immunoreactivity for Kv3.1b protein in the nucleus had been described before (Perney & Kaczmarek, 1997), we excluded this labeling from our analysis as we primarily focused on functional expression of potassium channels in the cell membrane.

### 3.1 Analysis of Kv3.1b expression in rat motor cortex

**Figure 2:**
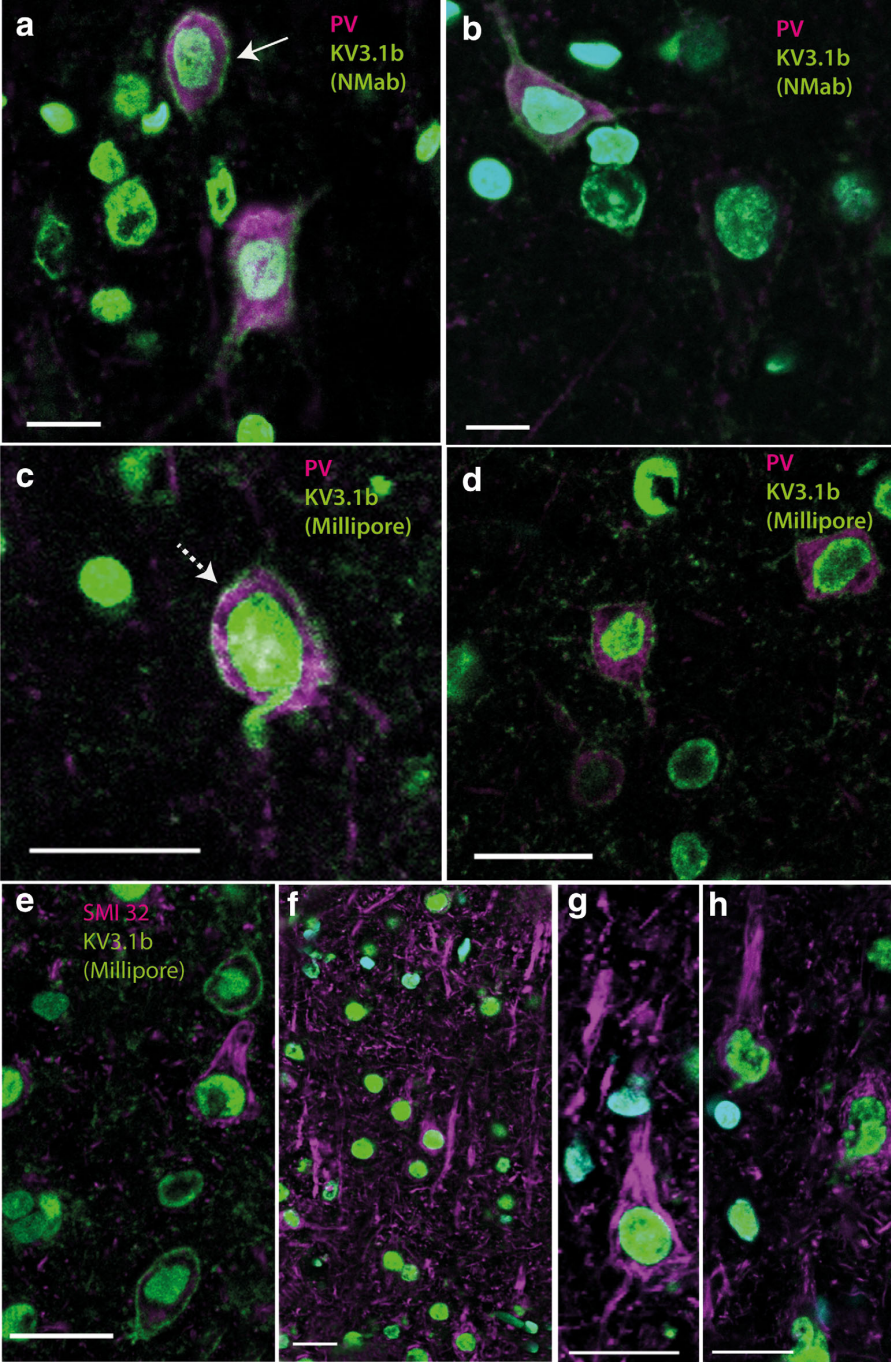
Kv3.1b expression in rat motor cortex. **T**ransverse sections of rat motor cortex labeled with Kv3.1b (green) antibodies and cell markers (magenta) as well as nuclear marker DAPI (blue). (a, b). The intensity of the membrane labeling of the neuron in a (solid arrow), which expressed Kv3.1b (NeuroMab, green) antibody and rabbit anti‐parvalbumin (magenta), is marked as “2A” in Figure 3b. (c, d) Kv3.1b (Millipore, green) antibody and mouse anti‐parvalbumin (magenta). (e–h) Examples of labeling for Kv3.1b (Millipore, green) antibody and mouse anti‐SMI32 (magenta). Note that neurons positively stained for SMI32 have pyramidal morphology but no clear Kv3.1b staining in the membrane. Scale bars: 20 µm [Color figure can be viewed at wileyonlinelibrary.com]

**Figure 3:**
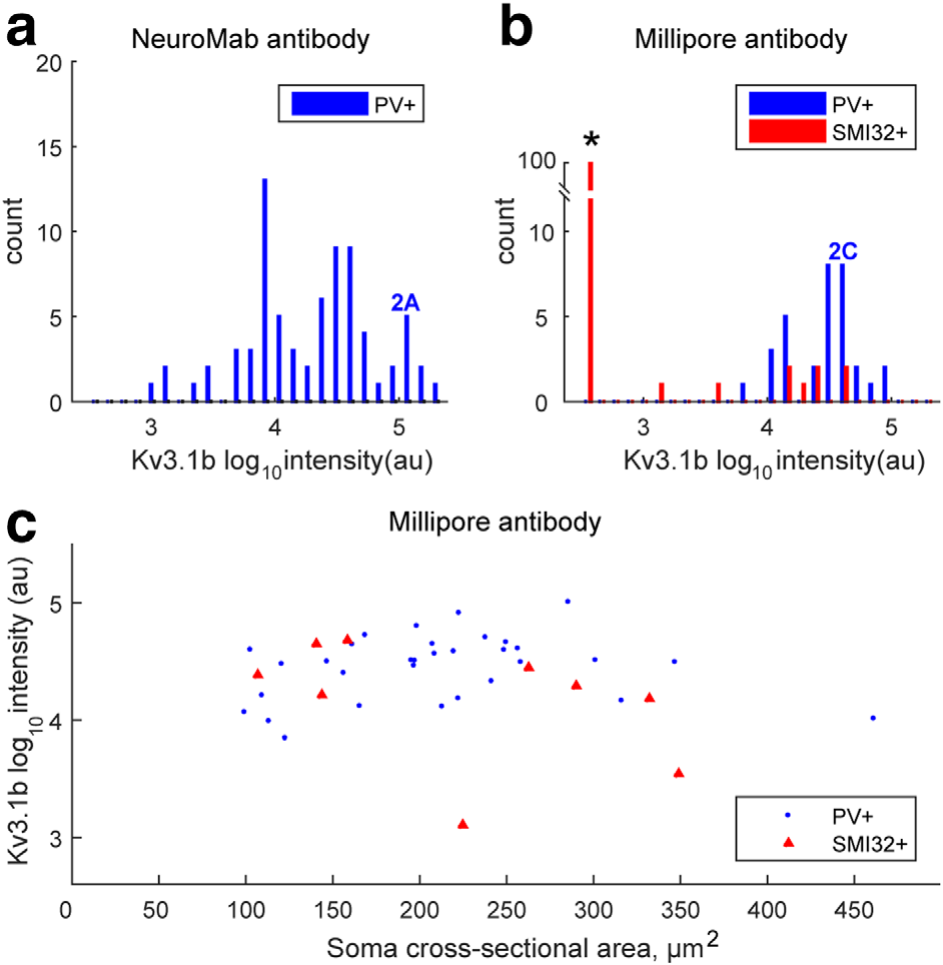
Quantification of Kv3.1b labeling in rat motor cortex. **T**he total intensity of Kv3.1b labeling in the membrane of each neuron was quantified using MetaMorph Offline software and expressed in arbitrary units (au). It was converted to a log_10_ scale. (a) Distribution of total intensities for staining with the NeuroMab antibody in 67 parvalbumin‐positive putative interneurons (PV+). The marker 2A indicates the intensity value for the PV + labeled neuron shown in Figure 2a. (b) The distribution of Kv3.1b staining intensity is shown for 32 neurons which stained with the Millipore antibody and were also parvalbumin‐positive (PV+) (blue bars). The marker 2C indicates intensity value for the PV + labeled neuron shown in Figure 2c. The red bars indicate Kv3.1b staining intensity for nine neurons which were also co‐labeled with the pyramidal cell marker SMI32. The other 91 neurons analyzed which expressed the SMI32 marker showed no Kv3.1v staining, and are indicated by the tall red column on the far left (*). (c) The intensity of Kv3.1b staining with the Millipore antibody has been plotted against the soma cross‐sectional area of each labeled cell for PV‐positive (blue circles) and SMI32‐positive neurons (red triangles). Only a few SMI32‐positive pyramidal cells expressed Kv3.1b [Color figure can be viewed at wileyonlinelibrary.com]

We first stained sections from rat motor cortex for both Kv3.1b antibodies and co‐labeled sections using parvalbumin or SMI32 antibodies (Figure 2). As expected, the NeuroMab Kv3.1b antibody stained neurons, some of which were also positive for parvalbumin and displayed interneuron morphology (Figure 2a,b). Analysis of Kv3.1b labeling intensity in the cell membrane of 74 parvalbumin‐positive cells taken from three rats revealed that almost all (67/74, 91%) parvalbumin‐positive cells expressed Kv3.1b, on the cell membrane at intensities above threshold. Figure 3a plots the frequency distribution of the Kv3.1b staining total intensities measured from parvalbumin‐positive cell membranes of these 67 cells, using the NeuroMab antibody. To give an indication of the intensity values plotted, the total intensity measure for the neuron highlighted in Figure 2a (solid arrow) is marked in the figure. As can be seen from Figure 3a, most of the neurons showed labeling in the higher range of intensities measured ( > 4 log au).

The Millipore Kv3.1b antibody also stained cell membranes of parvalbumin‐positive cells in the rat motor cortex (Figure 2c,d). We analyzed the total intensity of Kv3.1b labeling in the cell membranes of 32 neurons which were both parvalbumin‐positive and also clearly stained with the Millipore antibody. The intensity distribution is shown in Figure 3b (blue bars). The intensity measure of the Kv3.1b‐positive neuron identified in Figure 2c (dotted arrow) is marked in Figure 3b. Most labeled neurons expressed Kv3.1b in the same high intensity range as found with the NeuroMab antibody ( > 4 log au). The Kv3.1b‐positive neurons were generally small, with soma areas of 100–350 µm^2^ (Figure 3c).

Co‐labeling of the Millipore Kv3.1b antibody with the pyramidal cell marker SMI32 revealed that there was hardly any detectable Kv3.1b labeling present on pyramidal cell membranes, either in the cell bodies (Figure 2e–g) or apical dendrites (Figure 2f–h). In total, we examined 100 SMI32‐positive pyramidal neurons. Only nine of these cells showed any clear level of Kv3.1b expression in the cell membrane. The labeling intensity of these nine Kv3.1b‐positive cells is shown in Figure 3b,c (red symbols). The SMI32‐positive neurons which showed no Kv3.1b staining are shown in the tall red bar to the left of Figure 3b (*).

Thus, in the rat, the two different antibodies, raised in mouse and rabbit, indeed recognized the same membrane structures with very similar detection sensitivity, giving a consistent measure of the number of parvalbumin‐positive interneurons expressing Kv3.1b. In contrast, examination of the same cortical tissue showed that only a handful of SMI32‐positive pyramidal cells had any measurable expression of Kv3.1b.

### 3.2 Analysis of Kv3.1b expression in macaque motor cortex

**Figure 4:**
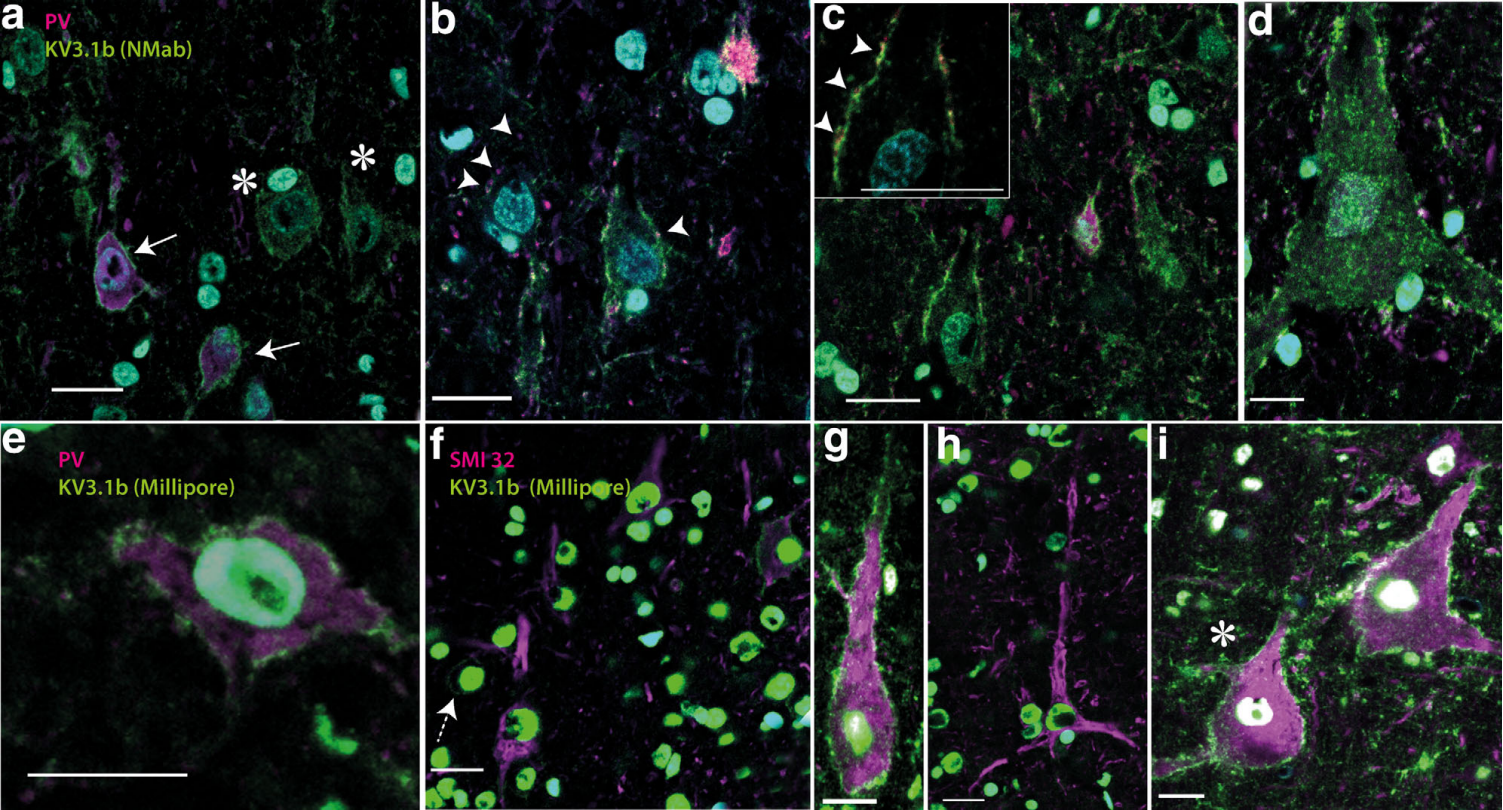
Kv3.1b expression in macaque motor cortex. **T**ransverse sections of macaque motor cortex labeled with Kv3.1b (green) antibodies and cell markers (PV and SMI32; magenta and DAPI in blue). (a–d) Kv3.1b (NeuroMab, green) antibody and anti‐parvalbumin (magenta). Parvalbumin‐positive neurons (solid arrows bottom left in (a) showed Kv3.1b membrane staining, but in addition, large, parvalbumin‐negative cells, with pyramidal cell morphology also strongly expressed Kv3.1b (asterisks). Further examples are shown in (b–d). High magnification revealed that pyramidal cells had numerous parvalbumin‐positive (magenta) synaptic boutons on their membranes (putative interneuron axon terminals; arrowheads in (b) and inset in (c), synaptic contact points shown as arrowheads), which contrasted with the Kv3.1b labeling present on these cells all along the membrane. (e–i) Show Kv3.1b labeling in neurons using the Millipore antibody (green) and either parvalbumin (e) or SMI32 antibody (f–i) (both magenta). The Millipore antibody stained the cell membrane in parvalbumin‐positive cells (e) but also labeled the membrane of large SMI32‐postitive pyramidal cells (g and i). (f) Shows four SMI32 positive pyramidal neurons, one of which (top right) is also positive for Kv3.1b. A small round SMI32‐negative neuron with clear Kv3.1b labeling is also present in this image (dashed arrow). (h) Shows another SMI32‐positive pyramidal cell that did not express Kv3.1b. Scale bars: 20 µm [Color figure can be viewed at wileyonlinelibrary.com]

We next analyzed sections obtained from macaque monkey motor cortex, to test whether both antibodies to Kv3.1b stained the membranes of parvalbumin‐positive interneurons reliably. Using the NeuroMab antibody, we found that the Kv3.1b staining pattern in parvalbumin‐positive cells was very similar to that described in rat tissues (Figure 4a). Thus, many parvalbumin‐positive cells in macaque cortex were small in size and had intense Kv3.1b labeling present on their cell membranes (Figure 4a, solid arrows).

Although co‐labeling of the NeuroMab Kv3.1b antibody with a specific pyramidal cell marker could not be carried out (see Methods), there were numerous parvalbumin‐negative cells that were positive for Kv3.1b and that bore the morphological hallmarks of pyramidal cells (Figure 4a, asterisks; Figure 4b–d). These Kv3.1b‐positive pyramidal cells measured up to 3,000 µm^2^ in cross‐sectional area, and included some very large cell bodies (Betz cells; Figure 4d). The larger pyramids were located in the deeper layers of the motor cortex and had their apical dendrite oriented perpendicular to the cortical surface. Kv3.1b labeling was clearly present in the membranes of both the soma and the apical dendrite (Figure 4a–d).

When parvalbumin immunostaining was found adjacent to pyramidal cell membranes (Figure 4b,c (inset, arrowheads), this was as discontinuous boutons scattered along the cell membrane, quite different to the Kv3.1b labeling which was present along the whole membrane. These parvalbumin‐positive boutons are likely to represent axon terminals of inhibitory interneurons on pyramidal cells (Chow et al., 1999).

**Figure 5:**
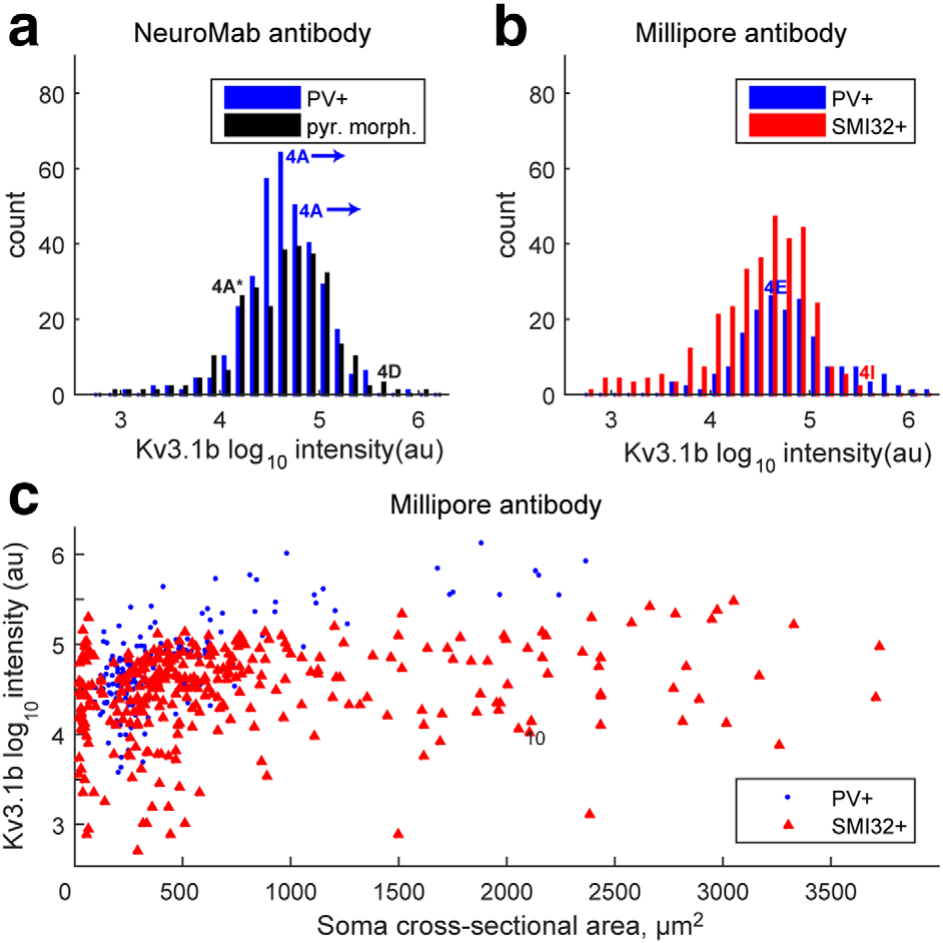
Quantification of Kv3.1b labeling in macaque motor cortex. (a). Distribution of total intensities for staining with the NeuroMab antibody in 347 parvalbumin‐positive putative interneurons (PV+, blue bars) in sections taken from macaque M1. The blue labels “4A” indicates the intensity of the two PV + labeled marked with arrows in Figure 4a. The black bars indicate the staining intensity of 282 neurons with pyramidal cell morphology. The black label 4A indicates the intensity value for the two pyramidal cells marked with * in Figure 4a (the values were very similar for the two cells). The value for another pyramid in Figure 3d is also marked. (b) Distribution of total intensities for staining with the Millipore antibody in 177 parvalbumin‐positive neurons (PV+) is shown as blue bars. The blue marker 4E indicates intensity value for the PV + labeled neuron shown in Figure 4e. The red bars indicate Kv3.1b staining intensity for 329 neurons colabeled with the pyramidal cell marker SMI32. The red marker 4I indicates intensity value for the SMI32‐labeled neuron shown in Figure 4i. Nearly all of the neurons expressing SMI32 showed clear Kv3.1b staining. Note the overlap in the range of Kv3.1b staining intensities in both a and b. (c) The intensity of Kv3.1b staining with the Millipore antibody has been plotted against the soma cross‐sectional area of each labeled cell for PV‐ (blue circles) and SMI32‐positive neurons (red triangles). Note the presence of a few very large pyramidal cells [Color figure can be viewed at wileyonlinelibrary.com]

Analysis of 347 parvalbumin‐positive putative interneurons in macaque M1 stained with the NeuroMab Kv3.1b antibody revealed that nearly all of them showed clear Kv3.1b labeling on their cell membranes (Figure 5a, blue bars). The Kv3.1b staining intensity measured in the two parvalbumin‐positive neurons marked by arrows in Figure 4a is indicated by “4A” (blue font and arrows) in Figure 5a.

In the same sections, stained for the NeuroMab Kv3.1b antibody, we found a population of 281 parvalbumin‐negative neurons with pyramidal cell morphology, almost all of which showed Kv3.1b staining (Figure 5a, black bars). Labeling intensity for NeuroMab Kv3.1b on the two pyramidal neurons (marked by asterisks) in Figure 4a was very similar. The intensity value for these cells is marked “4A” (black font and asterisk) in Figure 5a. The intensity of the large pyramidal cell shown in Figure 4d is also marked in Figure 5a. In general, there was a complete overlap in the range of NeuroMab Kv3.1b staining intensity for pyramidal neurons (black bars in Figure 5a) versus putative interneurons (blue bars).

Using the Millipore Kv3.1b antibody, we were able to further validate the presence of Kv3.1b in the membranes of 177 parvalbumin‐expressing neurons that were analyzed in macaque M1 (see example in Figure 4e). The distribution of the total intensity of staining in these neurons is shown in Figure 5b (blue bars; the total intensity of the neuron in 4e is indicated in the figure).

In the same Millipore Kv3.1b‐stained sections we found pyramidal cells that were SMI32‐positive, most of which also clearly expressed Kv3.1b on their cell membranes; again, both the soma and the apical dendrites were labeled (Figure 4g, i). A total of 329 pyramidal cells were identified and nearly all of these showed clear staining with Kv3.1b; their intensity distribution is shown in Figure 5b (red bars). The intensity value of the SMI32‐positive neuron identified by an asterisk in Figure 4i is indicated in Figure 5b. There was again a substantial overlap between the intensity of Millipore Kv3.1b staining for both putative interneurons (blue bars in Figure 5b) and pyramidal neurons (red bars in Figure 5b). A few SMI32‐positive neurons were clearly pyramidal in shape but did not express Kv3.1b (Figure 4f,h).

Figure 5c shows a plot of the intensity of Kv3.1b staining with the Millipore antibody against soma cross‐sectional area. SMI32‐positive pyramidal neurons showed a wide range of cross‐sectional areas and most were clearly labeled for Kv3.1b. Many macaque pyramidal neurons were larger than found in the rat (compare Figure 5c with 3c). All the large SMI32‐positive pyramidal neurones, including some very large ones with soma areas up to 3,500 µm^2^ (see Figure 4g,i), were strongly stained for Kv3.1b. Some very small SMI32‐positive pyramidal neurons (cross‐sectional areas less than 1,000 µm^2^) showed a wide range of Kv3.1b staining.

## 4 Discussion

In this comparative study, we characterized the expression pattern of the fast rectifier Kv3.1b potassium channel in motor cortex neurons, with particular attention to pyramidal cells. We found that most pyramidal neurons in macaque motor cortex express Kv3.1b labeling in the membrane of their cell bodies and apical dendrites, whereas this expression was very rarely found in rat motor cortex pyramidal cells positive for SMI32. It is well established that Kv3.1b is expressed in those rat cortical and hippocampal interneurons which also express parvalbumin (see Rudy & McBain, 2001) and this has been confirmed in the present study for both the rat and the macaque motor cortex.

We used two different antibodies (NeuroMab and Millipore) raised against different regions in the C terminal region of the Kv3.1b. To ensure that we analyzed only Kv3.1b immunoreactivity, one of our antibodies was raised using an amino acid sequence that is exclusively present on Kv3.1b and not Kv3.1a protein (Millipore antibody). The results indicate that both antibodies labeled the cell membranes of the same set of neurons. In both rat and macaque, the membranes of parvalbumin‐positive neurons were labeled with both antibodies (Figures 2 and 4). Both antibodies revealed labeling of cell membranes of many pyramidal‐shaped neurons in macaque cortex.

We quantified the extent of Kv3.1b expression in cell membranes of pyramidal neurons and parvalbumin‐positive interneurons. Although the expression pattern and intensity of Kv3.1b on the interneurons was similar in rat and macaque, there were marked differences in the expression pattern of Kv3.1b on the surface of pyramidal cells in rat versus macaque. Whereas we were only able to identify a few SMI32‐positive pyramidal cells in rat motor cortex that displayed any expression of Kv3.1b, we identified large numbers of macaque motor cortex pyramidal cells which were clearly stained with Kv3.1b. The data set included 281 pyramidal neurons immunostained with the NeuroMab antibody, and 329 pyramidal neurons double‐labeled with both the Millipore antibody and SMI32. Nearly all these neurons showed some expression of Kv3.1b (Figure 5a,b).

We used automated image analysis software to quantify the total intensity of all Kv3.1b stained regions of the membrane of the soma, plus the base of its apical dendrite. This involved a standard procedure applying the same threshold criteria for the creation of image masks that aided the selection of stained regions of cell membrane. We carried out an independent analysis for neurons stained with the two different antibodies. The quantification procedure must be treated with some caution, because we used the tyramide amplification system to detect Kv3.1b‐positive membranes with high sensitivity, and this results in a nonstoichiometric amplification of the signal (Matos, Trufelli, de Matos, & da Silva Pinhal, 2010; Stack, Wang, Roman, & Hoyt, 2014). Nevertheless, the results with the two different antibodies showed a consistent pattern across a large sample of stained neurons (Figure 5a,b). In particular, for both antibodies, the distribution of total Kv3.1b staining intensity was very similar in both pyramidal neurons and parvalbumin‐positive interneurons (Figure 5a,b).

The functional significance of the difference in Kv3.1b expression between rat and macaque pyramidal cells is intriguing. In the rodent, it is known that a family of Kv3 channels mediate fast rectifying potassium currents, which assist in the rapid repolarization of the cell after discharge of an action potential. Accordingly, the presence of Kv3.1b in parvalbumin‐positive interneurons (see e.g., Figure 2a,c,e) has been associated with the brief trough‐to‐peak duration (typically < 400 µs) of action potentials recorded extracellularly from some of these interneurons (Bartho et al., 2004; Contreras, 2004; Mountcastle, Talbot, Sakata, & Hyvarinen, 1969). These short‐duration “thin” spikes are thought to underlie the capacity for high‐frequency “fast spiking” in some of these interneurons (see Bean, 2007). Conversely, its absence from rodent pyramidal neurons is consistent with the long‐duration (typically ∼900 µs) spikes in these neurons, and their lower, regular spiking firing pattern (Bartho et al., 2004; Contreras, 2004; Hattox & Nelson, 2007). Thus in the rat, the duration of extracellularly‐recorded spikes is generally accepted as a robust means of discriminating interneurons from pyramidal cells (Bartho et al., 2004; Contreras, 2004). In this study, we confirmed that rat pyramidal neurons show very little evidence of Kv3.1b expression (Figures 2 and 3; Chow et al., 1999).

The situation in macaque motor cortex is clearly different, since Kv3.1b is present in most pyramidal neurons. The pattern of Kv3.1b expression in macaque M1 pyramidal neurons may be related to electrophysiological differences between rat and macaque pyramidal neurons. Thus, the widespread Kv3.1b expression among macaque M1 pyramids is consistent with the recordings of Vigneswaran et al. (2011) who found that 79% of antidromically identified pyramidal tract neurons (PTNs) in M1 had brief spike durations of < 400 µs; a few were even shorter than 200 µs. Thus for recordings in monkey cortex, action potential duration may be a less reliable means of distinguishing interneurons from pyramidal cells (Vigneswaran et al., 2011).

The electrophysiological subsample of M1 corticospinal neurons investigated by Vigneswaran et al. (2011) is of course not the same as the sample of pyramidal neurons identified immunohistochemically that is reported here. Corticospinal neurons represent only a small subset of all M1 pyramidal cells. However, Vigneswaran et al. (2011) also reported spike durations of “unidentified” neurons in M1 (those not responding antidromically to PT stimulation). Because of size bias in cortical recordings (see Firmin et al., 2014), these spikes are unlikely to have come from interneurons. Instead, these “unidentified neurons” were more likely to be other large M1 pyramidal cells projecting to different subcortical structures (corticostriatal, corticorubral, corticobulbar, and so forth), but not to the spinal cord. Importantly, most of these unidentified neurons also had short duration spikes (64% < 400 µs), again consistent with the Kv3.1b expression in many large pyramidal neurons reported here. A preliminary study of corticofugal pyramidal cells, identified from the cerebral peduncle, rather than from the PT, again found evidence for brief duration spikes (Lemon et al., 2012).

The range of pyramidal cell body size in macaque M1 is known to be much larger than in the rat (Donoghue & Kitai, 1981; Humphrey & Corrie, 1978; Landry, Wilson, & Kitai, 1984; Nudo, Sutherland, & Masterton, 1995) and this was consistent with our results. We measured the cross‐sectional area of each neuron analyzed (see Methods) and found that rat SMI32‐postive pyramids had cross‐sectional areas of up to 400 µm^2^, while most monkey SMI32‐positive pyramids were larger than this and ranged up to 3,500 µm^2^ (compare Figure 3c with Figure 5c).

We found that most of these larger macaque pyramids were clearly labeled for Kv3.1b, but there was no obvious relationship between the level of Kv3.1b expression and pyramidal cell size (Figure 5c). Given that the expression of membrane Kv3.1b is one of the factors contributing to short‐duration spikes, the finding that most of the larger pyramidal cells in the macaque motor cortex sections showed Kv3.1b labeling is consistent with brief spike duration in corticofugal pyramidal neurons (Lemon et al., 2012; Vigneswaran et al., 2011). However, these electrophysiological studies showed that not all large pyramidal neurons have brief spikes, and this might be related to the occasional finding in this study of macaque pyramids positive for SMI32 but negative for Kv3.1b (e.g., Figure 4h). What seems clear is that rat pyramidal cells, in contrast to those in the macaque, are characterized by small size, long duration spikes and an almost complete lack of Kv3.1b expression.

It is unknown whether, in the macaque, spike duration is more generally linked to other neuronal properties including axonal size, conduction velocity and firing patterns (Bean, 2007; Firmin et al., 2014; Perge, Niven, Mugnaini, Balasubramanian, & Sterling, 2012). Other fast potassium channels, such as Kv2, may also contribute to spike properties (Bean, 2007; Guan, Armstrong, & Foehring, 2013; Porcello, Ho, Joho, & Huguenard, 2002).

These differences may be related to other contrasting features of the rat versus macaque descending pathways (Lemon, 2008). In the rat, pyramidal cells that give rise to the corticospinal tract have relatively slow conduction velocities, with even the fastest axons conducting at < 20 m.s^−1^ (Mediratta & Nicoll, 1983). In contrast, one of the characteristic features of the macaque motor system is the presence of large corticospinal neurons in the motor cortex which have very fast axons, conducting up to 80–90 m.s^−1^ (Firmin et al., 2014; Porter & Lemon, 1993). These fast‐conducting neurons may be of particular functional significance because many of them make direct monosynaptic connections with alpha motoneurons of limb muscles, and particularly with those innervating the most distal muscles moving the fingers and toes (Lemon, 2008; Rathelot & Strick, 2006). These excitatory cortico‐motoneuronal connections are unique to certain, dexterous primate species.

## 5 Conclusion

This study has revealed a major species difference in the occurrence of the fast Kv3.1b potassium channel in rat versus macaque monkey motor cortex pyramidal neurons. The significance of this difference requires further investigation, and particularly in pyramidal neurons with identified target structures. For the corticospinal projection, it could be related to electrophysiological differences, including spike duration and firing rate, but might also reflect the presence, in the macaque, of motor cortex corticospinal neurons with fast conducting axons which are absent in the rat motor cortex. Further research is needed to explain these intriguing species‐specific differences.

## Conflict of interest

The authors declare no conflicts of interest.
